# Target-induced hot spot construction for sensitive and selective surface-enhanced Raman scattering detection of matrix metalloproteinase MMP-9

**DOI:** 10.1007/s00604-024-06183-w

**Published:** 2024-01-19

**Authors:** Huihui Jin, Tianqing Liu, Dan Sun

**Affiliations:** 1 School of Pharmacy, Nantong University, Nantong, 226001 Jiangsu China; 2 NICM Health Research Institute, Western Sydney University, Westmead, NSW 2145 Australia

**Keywords:** Surface-enhanced Raman scattering, Gold nanosphere, Matrix metalloproteinase-9, Hot spot effect

## Abstract

Studies have found that matrix metalloproteinase-9 (MMP-9) plays a significant role in cancer cell invasion, metastasis, and tumor growth. But it is a challenge to go for highly sensitive and selective detection and targeting of MMP-9 due to the similar structure and function of the MMP proteins family. Herein, a novel surface-enhanced Raman scattering (SERS) sensing strategy was developed based on the aptamer-induced SERS “hot spot” formation for the extremely sensitive and selective determination of MMP-9. To develop the nanosensor, one group of gold nanospheres was modified with MMP-9 aptamer and its complementary strand DNA1, while DNA2 (complementary to DNA1) and the probe molecule 5,5′-dithiobis-(2-nitrobenzoic acid) (DTNB) were grafted on the surface of the other group of gold nanospheres. In the absence of MMP-9, DTNB located on the 13-nm gold nanospheres has only generated a very weak SERS signal. However, when MMP-9 is present, the aptamer preferentially binds to the MMP-9 to construct MMP-9–aptamer complex. The bare DNA1 can recognize and bind to DNA2, which causes them to move in close proximity and create a SERS hot spot effect. Due to this action, the SERS signal of DTNB located at the nanoparticle gap is greatly enhanced, achieving highly sensitive detection of MMP-9. Since the hot spot effect is caused by the aptamer that specifically recognizes MMP-9, the approach exhibits excellent selectivity for MMP-9 detection. Based on the benefits of both high sensitivity and excellent selectivity, this method was used to distinguish the difference in MMP-9 levels between normal and cancer cells as well as the expression of MMP-9 from cancer cells with different degrees of metastasis. In addition, this strategy can accurately reflect the dynamic changes in intracellular MMP-9 levels, stimulated by the MMP-9 activator and inhibitor. This strategy is expected to be transformed into a new technique for diagnosis of specific cancers related to MMP-9 and assessing the extent of cancer occurrence, development and metastasis.

**Graphical Abstract:**

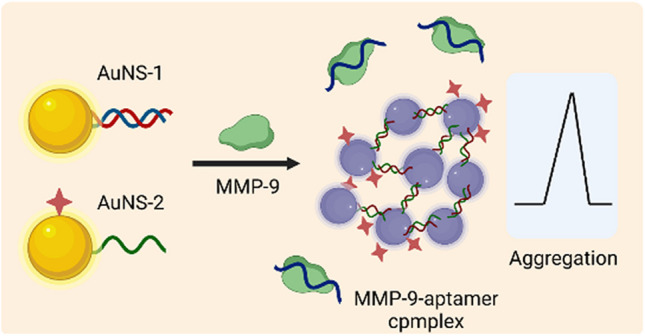

**Supplementary Information:**

The online version contains supplementary material available at 10.1007/s00604-024-06183-w.

## Introduction

Matrix metalloproteinases (MMPs) as a family of zinc-dependent endopeptidases have specific proteolytic ability for several substrates across the extracellular matrix (ECM) [1, 2]. More importantly, MMP-9 serves as a significant role in membrane protein cleavage and ECM remodeling [3]. It has been discovered that MMPs’ overexpression and activation are strongly associated to a variety of cancers [4–6]. MMP-2 and MMP-9 are overexpressed in breast cancer [7], cervical cancer, etc. Moreover, the prognostic value of MMPs has also been well demonstrated in treatment of different cancers [8, 9]. For example, patients with high levels of MMP-2 and MMP-9 were clinically defined as a poor prognosis [10]. MMP-9 is therefore introduced as a possible biomarker in light of its potential application in diagnosis and treatment monitoring of tumors’ progression [11]. The highly sensitive and selective determination of MMPs is important for elucidating the mechanism of disease occurrence and developing cancer diagnosis and treatment strategies at early stage. However, it is a challenge to go for highly sensitive and selective detection and targeting of MMP-9 due to the similar structure and function of the MMP proteins family [12].

To date, there have been a number of reported MMP-9 detection methods including immunohistochemistry approaches [13], enzyme-linked immunosorbent assay (ELISA) [14], fluorescence resonance energy transfer (FRET) [15], electrochemical assay [16], surface plasmon resonance (SPR) assays [17], and near-infrared (NIR) fluorescent methods [18]. The traditional ELISA assay has been widely adopted as the gold standard. However, common drawbacks including tedious and time-consuming operational steps and expensive costs severely limit the development of the method. To overcome these issues, FRET approaches were developed as possible replacements, and the results were astounding. Unfortunately, defects such as photobleaching and phototoxicity have prevented its progress.

More recently, surface-enhanced Raman spectroscopy (SERS) has been evolved into a prominent bioanalytical strategy due to its ability to give out abundant fingerprint information of important biological molecules from living cells and tissues [19–24]. This technique has significant advantages for biochemical applications, including ultra-high sensitivity, multiplex detection with high spectral resolution, selectable excitation wavelengths, non-invasiveness to biological samples, and, especially, resistance to photobleaching and autofluorescence, when compared to other methods. These distinct advantages make SERS an excellent technique for the detection of biological samples and live cells imaging [25].

The “hot spot” model that exhibits excellent SERS activity has attracted great attention for trace-molecule detection [26, 27]. The great SERS enhancement can be generated in gaps when two nanoparticles are close enough to each other. Such spatial multi-local locations with extremely high electric field enhancement and strong SERS signal are called hot spots [28]. Hot spots usually exist in the gaps between nanoparticle aggregates. The size of the gap is directly crucial to the construction of the “hot spot” [29–31], and even tiny changes at the nanoscale can significantly alter the performance of SERS. In addition, another key element is that the probed molecules are required to be placed precisely in the gaps in order to achieve the proper and strong response from the probed matters. Therefore, in order to maximize the sensitivity of SERS bioassays, there is a great need to develop effective SERS substrates with nanostructured coupling. Among them, the use of targeted molecules (e.g., antibody-antigen [32, 33], protein [34], aptamer [35], and DNA [36]) to induce aggregation or de-aggregation of nanoparticles is a common strategy to modulate the SERS signal.

**Scheme 1:**
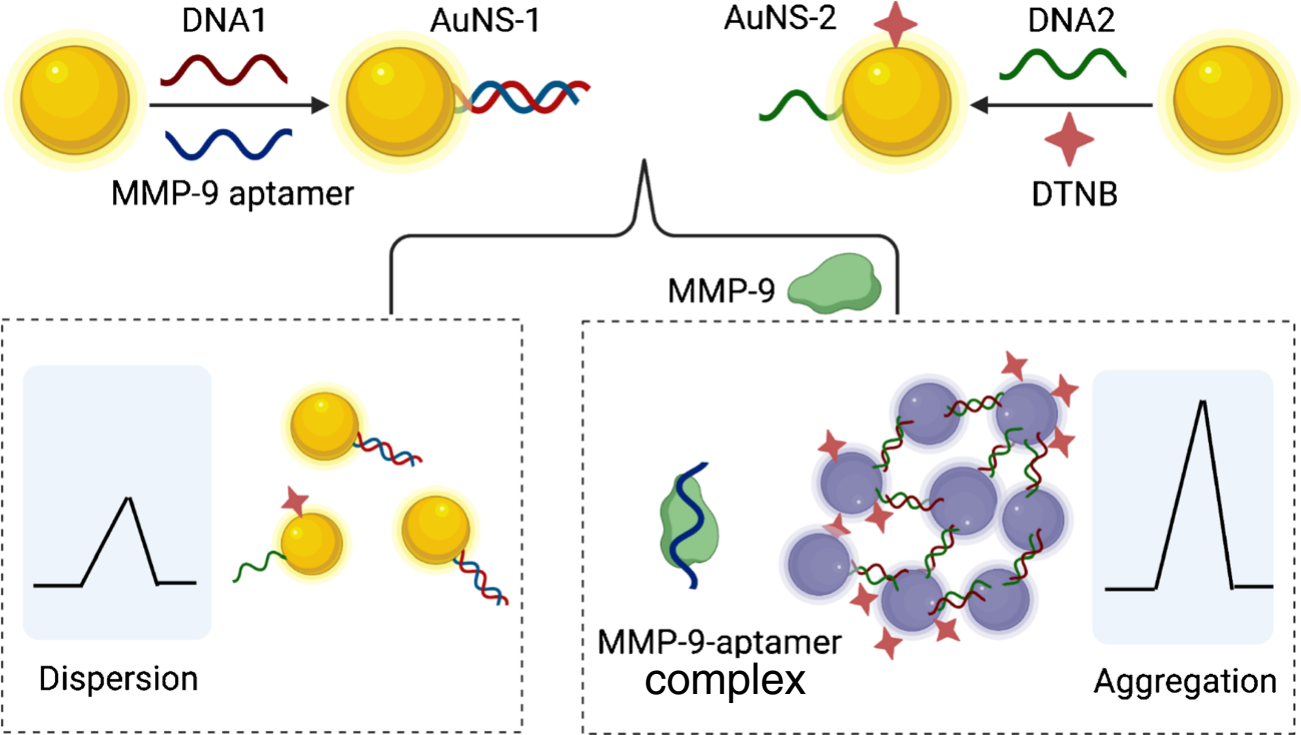
Illustration of the SERS detection of MMP-9 through the formation of “hot spot” effect induced by the target

Herein, we developed a SERS off-turn strategy for detecting cellular MMP-9 based on the target-triggered information of hot spots (Scheme 1). Gold nanosphere-1 (AuNS-1) and gold nanosphere-2 (AuNS-2) were prepared as two important components of the SERS nanosensor by modifying the DNA strands on the AuNSs. The MMP-9 aptamer and its partial complementary sequence (DNA1) were decorated on the surface of AuNSs to form AuNS-1. For AuNS-2, DNA2 (complementary to DNA1) and the probe molecule DTNB were conjugated on the nanoparticle surface. When encountering MMP-9, MMP-9 aptamer prefers to bind to MMP-9 to form a complex compared to DNA1. The released DNA1 is able to bind to DNA2 allowing AuNS-1 and AuNS-2 to couple together. The coupling of AuNS-1 and AuNS-2 results in the aggregation of the AuNSs and the information of the hot spots, which produces an obvious SERS intensity change of DTNB. Based on this approach, the MMP-9 levels in healthy and malignant cell lines were evaluated, and different cancer cell metastasis capacities were also compared, which has rarely been detected in previous investigations.

## Results and discussion

### Characterization of nanosensors and performance in response to MMP-9

In order to reduce the non-specific adsorption of impurity proteins on the surface of AuNSs, we optimized the amount of DNA strand coverage on the surface of AuNSs by adjusting the content ratio between AuNSs and DNA strand. The results are shown in Fig. S1. For AuNS-1, when the molar ratio of AuNSs:duplex is set at 1:60, the amount of DNA covering on the gold sphere is the largest, and it is practically same at 1:80. Therefore, we modify the corresponding DNA1/aptamer duplexes on the gold sphere according to AuNSs:duplex which is 1:60. For AuNS-2, the amount of DNA coverage on the gold sphere reached its maximum when AuNSs:single strand was 1:100, so this ratio was selected as the reference basis for preparing AuNS-2.

**Fig. 1:**
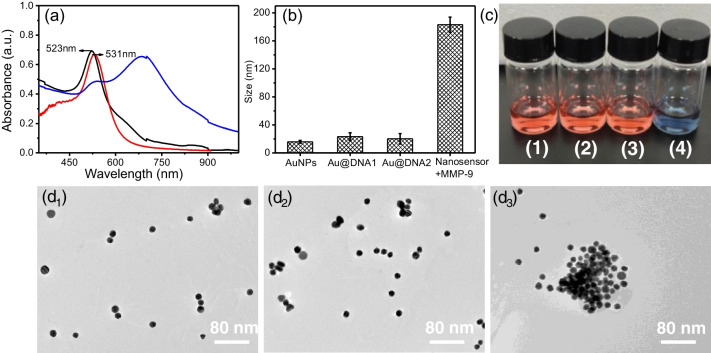
(**a**) UV–vis spectra of AuNPs (black line), Au@DNA1 (red line) and the nanosensor with MMP-9 (blue line). (**b**) Hydrodynamic size and (**c**) color changes of the AuNPs (1), Au@DNA1 (2), Au@DNA2 (3), and the nanosensor incubated with MMP-9 (4). TEM images of the AuNPs (d_1_), Au@DNA1 (d_2_), and the nanosensor incubated with MMP-9 (d_3_)

To verify the response of this nanosensor to MMP-9, ultraviolet (UV) absorption spectra and dynamic light scattering (DLS) as well as transmission electron microscopy (TEM) of the gold nanoparticles and nanosensor were performed before and after encountering MMP-9 (350 ng/mL). As shown in Fig. 1(a), the UV maximum absorption wavelength of the synthesized gold nanoparticles is 523 nm, which corresponds to an average particle size of approximately 13 nm (Fig. 1(d_1_)). Gold nanoparticles of this size have almost no Raman enhancement effect with laser wavelength 785 nm, which provides a larger response range for the SERS signal enhancement generated by MMP-9-induced gold nanosphere aggregation, and the resulting change in relative signal intensity allows a more sensitive detection. The maximum absorption wavelength of the gold nanospheres was red-shifted to 531 nm after DNA fixation, but the peak shape did not broaden, indicating that the DNA modification had no impact on the stability of gold nanoparticles in aqueous solution. The absorption peak of the gold nanospheres broadened and underwent a significant red-shift when the nanosensor encountered the target MMP-9, demonstrating that MMP-9 caused the aggregation of gold nanoparticles. It was also evident in the fact that the hydrodynamic size in DLS significantly increased when the nanosensor came into contact with MMP-9 (Fig. 1(b)). To further demonstrate the capacity of MMP-9 to promote gold nanoparticle aggregation, we compared the optical photographs of AuNSs, DNA-modified AuNSs, and the nanosensors in solution following reaction with MMP-9. As shown in Fig. 1(c), the prepared AuNSs had a distinct bright red wine color and were able to maintain the same color after modifying DNA1 and DNA2, indicating that the DNA modification did not destabilize the gold nanospheres. However, when the nanosensor encountered MMP-9, the barely leaked DNA1 was able to complementarily combine with DNA2 because MMP-9 had the capacity to competitively bind to its aptamer. The gold nanospheres attached to the ends of DNA1 and DNA2 caused the solution to change color from wine red to blue in the aggregated state. Additionally, the TEM images offered a clearer visual representation of the MMP-9-induced aggregation of AuNSs (Fig. 1(d_3_)). We have compared the number of hot spots formed between gold nanoparticles at various MMP-9 doses. As shown in Fig. S2, when MMP-9 concentration increased, more and more hot spot structures were formed between the golden spheres, which was beneficial to the enhancement of SERS signal of probe molecules, enabling very sensitive detection of MMP-9.

### Sensing application of the SERS off-turn strategy

**Fig. 2:**
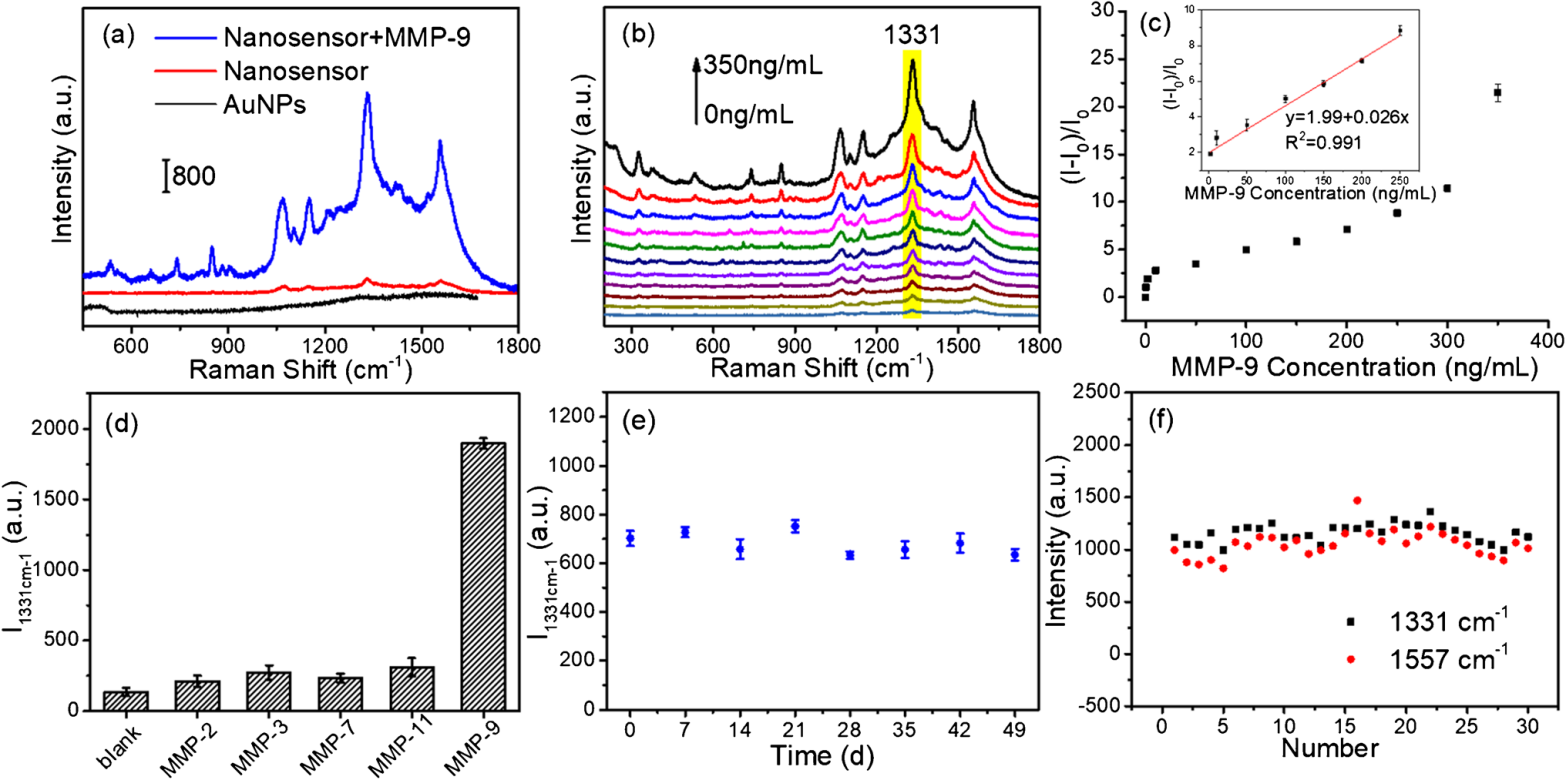
**a** SERS spectra of the AuNPs, nanosensor without/with MMP-9 (350 ng/mL). **b** SERS spectra of the nanosensor with different concentrations of MMP-9 (from the bottom to the top 0, 0.2, 2, 10, 50, 100, 150, 200, 250, 300, 350 ng/mL). **c** The (*I* − *I*_0_)/*I*_0_ value of DTNB *vs* the concentration of MMP-9. **d** SERS response of 1331 cm^−1^ to other interferents (100 ng/mL) and MMP-9 (10 ng/mL). **e** SERS intensity change of DTNB (1331 cm^−1^) located on the nanosensor under different storage time lengths. **f** The signal intensities of 1331 cm^−1^ and 1557 cm^−1^ at 30 points

Based on the aforementioned MMP-9-induced gold nanoparticle aggregation, we further validated the performance of this SERS nanosensor to detect MMP-9 in aqueous solution. The SERS spectra of AuNSs and the nanosensor before and after being exposed to MMP-9 were compared. Figure 2a shows that the gold nanoparticles themselves had no Raman signal and did not affect the determination of the target. The nanosensor merely emited a very weak SERS signal of the probe molecule DTNB when it did not interact with MMP-9. However, the SERS signal of DTNB was significantly enhanced when it reacted with MMP-9. The hot spot effect formed by the MMP-9-induced aggregation of gold nanoparticles produced an amplified electromagnetic field and served to improve the detection sensitivity.

Condition optimization for quantifying MMP-9 level was carried out. The incubation time for SERS intensity of the nanosensors at 1331 cm^−1^ (the symmetric stretch for the nitro group of DTNB) in the presence of MMP-9 (10 ng/mL) was assessed in Fig. S3a. It is noted that SERS intensity at 1331 cm^−1^ is sharply increased within the first 120 min. When the reaction time is 120 min, the SERS intensity of 1331 cm^−1^ reaches the maximum value (Fig. S3b). Thus, 120 min is chosen as the optimum incubation time. The intensity of SERS signal of the nanosensor was investigated at various temperature and pH conditions. Fig. S4b illustrates the effect of temperature values ranging from 20 to 45℃ on the SERS signal intensity produced by MMP-9 (100 ng/mL) with the greatest SERS intensity at 1331 cm^−1^ occurring at 35℃. As a result, we selected 35℃ as the optimum temperature. As shown in Fig. S5a, the SERS intensity increased as the pH value increased from 5.5 to 8.5, reached a maximum at 7.5, and then progressively decreased when the pH value was raised further. Thus, 7.5 was chosen as the optimum pH value.

Furthermore, the sensing performance using different concentrations of MMP-9 aqueous solutions was measured. As shown in Fig. 2b, the SERS signal of DTNB located in the gold nanoparticles was gradually enhanced as the concentration of MMP-9 increased. This suggests that the high concentration of MMP-9 induced the formation of more hot spot structures between the gold nanoparticles, resulting in a significant enhancement of the SERS signal of DTNB located in the nano-gaps. The rate of change of the signal intensity of DTNB at 1331 cm^−1^ ((*I* − *I*_0_)/*I*_0_) presented a good linear relationship with the MMP-9 concentration (2–200 ng/mL). *I*_0_ and I respectively represent the SERS intensities of DTNB at 1331 cm^−1^ before and after the reaction at different concentrations of MMP-9. The linear equation was *y* = 1.99 + 0.026 × with a correlation coefficient of 0.991. The limit of detection (LOD) was calculated as 0.2 ng/mL, which is lower than other methods that have been reported (Table S1). The detection limit is determined by the lowest concentration that can be detected at a signal-to-noise ratio of 3:1.

Selectivity, stability, and reproducibility are important indicators to evaluate the detection performance of a nanosensor. As shown in Figs. 2d and S6, the other MMP proteins had no effect on the SERS signal enhancement of the probe molecule, proving that these substances did not bind to the aptamer of MMP-9. In contrast to these MMP proteins, MMP-9 was able to significantly increase the SERS signal of DTNB. This demonstrates that the nanosensor has a high selectivity for the specific detection of MMP-9. In addition, we also evaluated the interference of other small biomolecules in the determination of MMP-9 in Fig. S7. Only MMP-9 induced significant Raman signal changes, suggesting negligible interference from the other biological molecules. To verify the stability of the nanosensor, the constructed nanosensor was placed at room temperature for varying lengths of time, and then SERS spectra of DTNB located on the nanosensor were collected every week (Fig. S8). The nanosensor showed obtained almost uniform amplification after being in place for various time periods for the response to the same concentration of MMP-9 (Fig. 2e), which indicates that the nanosensor had excellent stability. We further compared the SERS signal intensity of the probe molecule DTNB on AuNS-2 over time when the molar ratio of AuNSs:single is 1:25, 1:50, and 1:100 (Fig. S9). The results show that the SERS signal intensity of DTNB in this case of 1:100 almost remains constant with the prolongation of the placement time. However, 1:25 displayed a trend of signal enhancement at a very early stage, which was attributed to the fact that the surface of the gold spheres was not completely covered with DNA. As a result, there was the non-specific adsorption of impurity proteins on the surface of the gold spheres, which prompted the aggregation of nanoparticles and improved the SERS signals. An enhancement of the signals at an intermediate time was visible at 1:50. According to the aforementioned findings, the molar ratio of 1:100 for AuNSs:single maximized the stability of gold nanoparticles in solution. After the nanosensor reacted with MMP-9, we randomly selected any 30 positions on the same nanosensor and measured their SERS spectra (Figs. 2f and S10). The signal intensities at 1331 and 1557 cm^−1^ (aromatic ring mode) were used to depict these 30 positions. We found that the variation in signal intensity between the points was essentially inconsequential, and the relative standard deviations (RSDs) of Raman signals are calculated as 4.52% and 5.84% at 1331 and 1557 cm^−1^. This indicates that this SERS nanosensor has good detection reproducibility.

To demonstrate the potential practicality of our nanosensor, we employed it to determine MMP-9 in real samples (human serum sample) by addition standard method (Table S2). We can find the recovery rate of MMP-9 range from 92 to 106%, which proves that our nanosensor is practical for real samples and suitable for the early diagnosis of MMP-9 infections. Furthermore, we compared its analytical performance with that of a commercially available ELISA. Table S3 demonstrated that there is no signification difference between the results given by the two methods. As a result, the proposed SERS sensing strategy could be reasonably applied in the determination of MMP-9.

### Differentiation of MMP-9 levels in normal and cancer cells

**Fig. 3:**
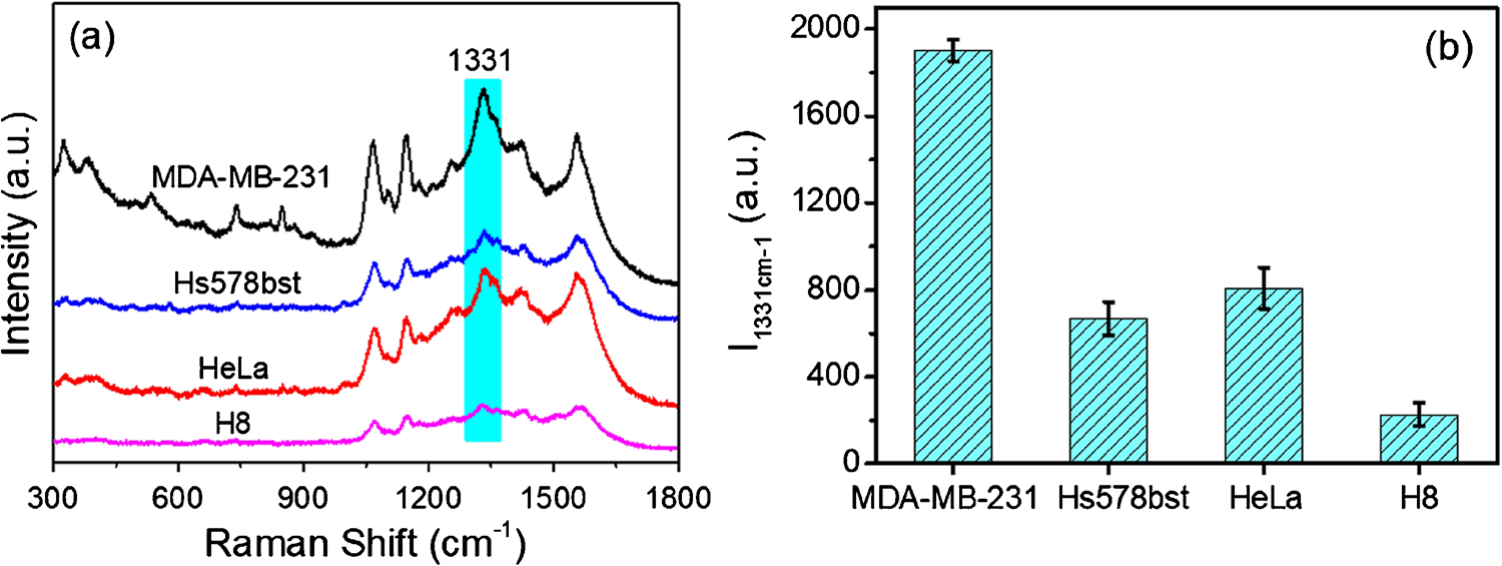
**a** Comparison of SERS spectra of normal cells (H8, Hs578bst) and cancer cells (HeLa, MDA-MB-231). **b** Corresponding histogram of *I*_1331 cm−1_ along with different cells

Based on the excellent performance of this nanosensor in detecting MMP-9 in aqueous solution, we subsequently employed it to evaluate the difference in the level of secreted MMP-9 between normal and cancer cells to reveal the feasibility of MMP-9 as a tumor marker in early disease diagnosis. Two groups of cancer cells with different levels of metastasis and their corresponding normal cells were incubated for 24 h in a culture dish. The supernatants of the four groups each received the same quantity of the nanosensor, and the SERS spectra of each group were measured after 2 h reaction. Figure 3a shows that the SERS spectrum for the MDA-MB-231 cells with high metastasis was significant, while the SERS signal of corresponding normal breast cells was modest, indicating that cancer cells expressed more MMP-9 than normal cells. The other group of cells (HeLa) also showed a similar trend. This tentatively suggests that it is possible to use MMP-9 as a biomarker for early diagnosis of some specific cancers according to the difference in MMP-9 expression levels in normal and cancer cells.

### Comparison of MMP-9 levels in different metastasis degrees of cancer cells

**Fig. 4:**
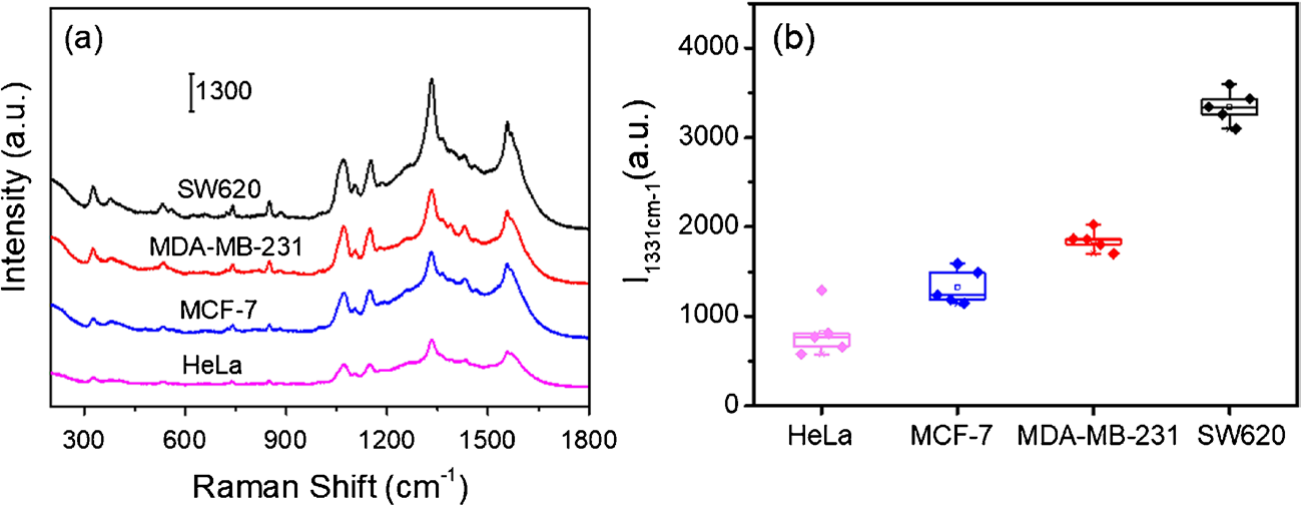
**a** Comparison of SERS spectra of cancer cells with different levels of metastasis. **b** Corresponding box-and-whisker plots of *I*_1331 cm−1_ along with different cells, *n* = 5

Studies have shown that MMP-9 is associated with the progression of cancer development and metastasis. The expression levels of MMP-9 secreted by several cancer cells with varying levels of metastasis were compared using our nanosensors. The SERS spectral signals for SW620 cells and MDA-MB-231 cells with the higher degree of metastasis were noticeably stronger in comparison to cells with lower degrees of metastasis (Fig. 4a), suggesting that cancer cells with higher degrees of metastasis secreted more MMP-9, which is consistent with the findings reported in the literature. Among them, the SERS signal of SW620 cells was the strongest, corresponding to the highest MMP-9 secretion level (Fig. 4b). However, HeLa cells had the lowest MMP-9 expression and the weakest SERS signal of all the cell types. The above results indicate that the constructed SERS nanosensor can sensitively monitor the MMP-9 levels in different cancer cells and provide a reliable reference strategy for determining the extent of cancer cell metastasis.

### SERS monitoring of dynamic changes of MMP-9

**Fig. 5:**
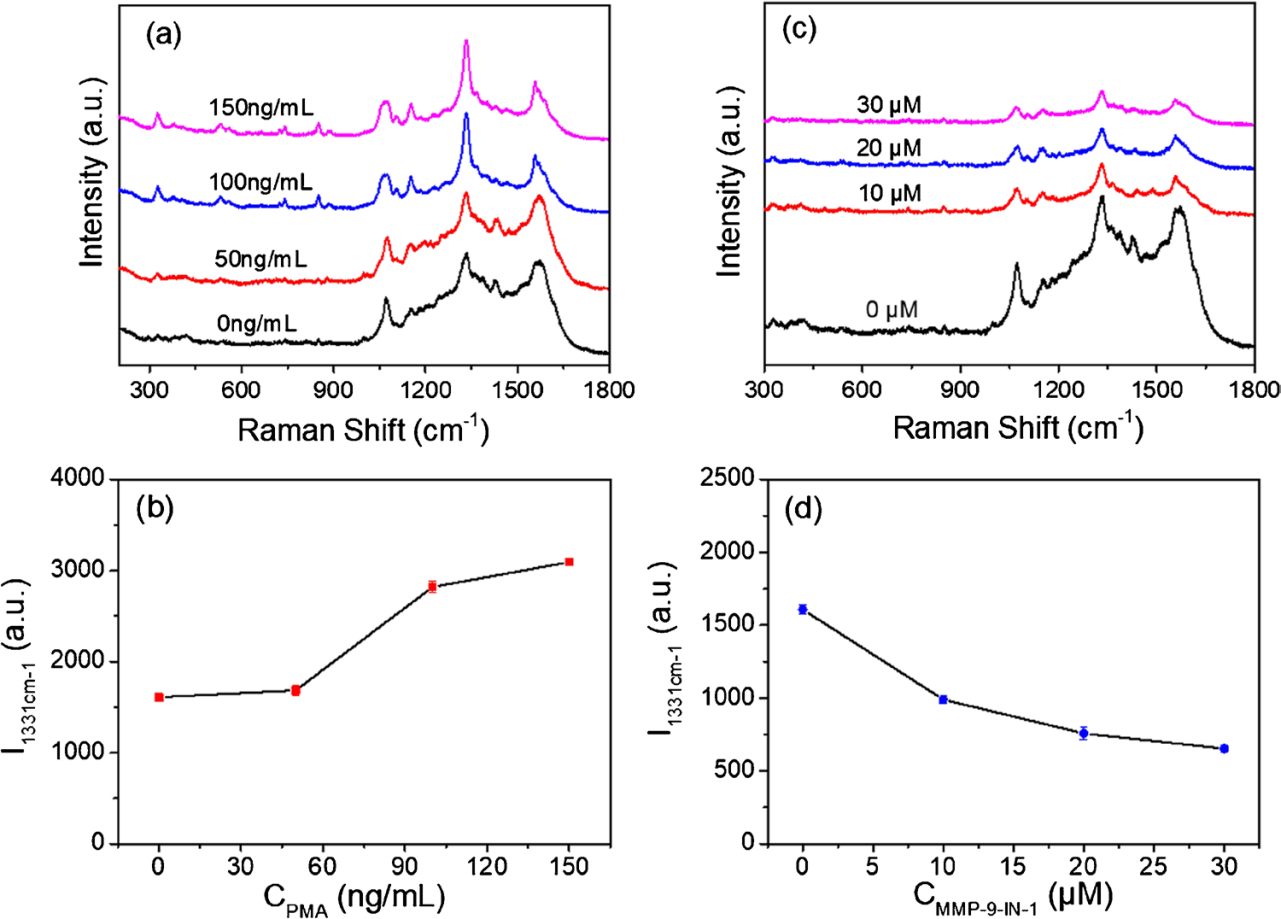
The SERS spectra of MDA-MB-231 cell after incubation with different concentrations of inducer PMA (**a**) and inhibitor MMP-9-IN-1 (**c**). Corresponding broken line graph of *I*_1331 cm−1_ along with different concentrations of PMA (**b**) and MMP-9-IN-1 (**d**)

The expression of intracellular MMP-9 can be regulated by MMP-9 inducers and inhibitors. To trace this dynamic change in MMP-9 levels, we treated MDA-MB-231 cells with inducer phorbol 12-myristate 13-acetate PMA (different concentrations) [37] for 4 h and then monitored their SERS spectra. We found that the SERS signal of DTNB was continuously enhanced with increasing inducer concentrations (Fig. 5a, b), indicating that PMA was able to stimulate MDA-MB-231 cells to produce more MMP-9. Furthermore, inhibitor MMP-9-IN-1 [38] was used to treat MDA-MB-231 cells. MMP-9-IN-1 did have an inhibitory effect on the ability of MDA-MB-231 cells to express MMP-9, as evidenced by the fact that the SERS signal of DTNB was consistently diminished as the inhibitor concentration increased (Fig. 5c, d). The above experimental results indicate that this SERS nanosensor can monitor the dynamic changes of intracellular MMP-9 levels, which provides strong support for the continuous tracking of the cancer development process.

## Conclusions

In summary, the SERS sensing platform based on nano-aggregates was developed for the intracellular MMP-9 detection via a target-triggered hot spot effect. In the presence of the MMP-9, MMP-9 aptamer prefers to bind to MMP-9 to form a complex compared to DNA1. The released DNA1 is able to bind to DNA2 on AuNPs-2 to induce coupling between AuNPs-1 and AuNPs-2, resulting in the formation of SERS hot spot that improves the SERS signal of probe DTNB. With this design, the MMP-9 levels in cancer and normal cells, as well as in various metastasis stages, were evaluated, revealing that the cellular MMP-9 in cancer cells display a higher content than normal cells, and the levels in high malignant SW620 and MDA-MB-231 cells are higher than those in low malignant MCF-7 and HeLa cells. Moreover, this SERS nanosensor was capable of monitoring the dynamic changes of intracellular MMP-9 levels stimulated by MMP-9 inducers and inhibitors. We believe that this approach can open new avenues for MMP-9-based biomedical detection at the early cancer stage and provide guidance for pathophysiological mechanism studies, cancer diagnosis, and therapy.

### Supplementary Information

Below is the link to the electronic supplementary material.

Supplementary file1 (DOCX 542 KB)
