# Supplementary material for: Target-induced hot spot construction for sensitive and selective surface-enhanced Raman scattering detection of matrix metalloproteinase MMP-9
Source: Mikrochim Acta. 2024 Jan 19;191(2):105. doi: 10.1007/s00604-024-06183-w (PMC10798921; doi:10.1007/s00604-024-06183-w)
Supplement: Supplementary file 1 — Supplementary file1 (DOCX 542 KB) [file 604_2024_6183_MOESM1_ESM.docx]

**Supporting information**

**Target-induced hot spot construction for sensitive and selective surface-enhanced Raman scattering detection of matrix metalloproteinase MMP-9**

Huihui Jin^a^, Tianqing Liu^b^*, Dan Sun^a^*

^a^ School of Pharmacy, Nantong University, Nantong, Jiangsu 226001, China

^b^ NICM Health Research Institute, Western Sydney University, Westmead, Australia

∗ Corresponding author.

E-mail addresses: dsun1203@ntu.edu.cn (D. Sun)

E-mail addresses: michelle.tianqing.liu@gmail.com (T. Liu)

1. **Experimental section**

**1.1 Reagents and instruments**

Gold(III) chloride trihydrate, trisodium citrate (98%), magnesium chloride (MgCl_2_), sodium chloride (NaCl) and tris(2-carboxyethyl)phosphine hydrochloride (TCEP) were obtained from Aladdin Industrial Corporation (Shanghai, China). 5,5’-Dithiobis-(2-nitrobenzoic acid) (DTNB), phorbol 12-myristate 13-acetate (PMA, ≥99%) and inhibitor MMP-9-IN-1 were purchased from Sigma-Aldrich. Human recombinant MMP-9, MMP-2, MMP-3, MMP-7 and MMP-14 were purchased from Thermo Fisher Scientific. The certified fetal bovine serum was purchased from VivaCell. The sequences of the DNA strands are the following: MMP-9 aptamer: 5’-TCG TAT GGC ACG GGG TTG GTG TTG GGT TGG-3’. DNA1: 5’-GGT GTG CCA TAC GAA A/3ThioMC3-D/-3’. DNA2: 5’-TCG TAT GGC ACA CCA A/3ThioMC3-D/-3’, where /3ThioMC3-D/ stands for 3-(butyldisulfaneyl) propan-1-ol to provide a disulfide structure at the 3’ end of the DNA strands.

The morphologies of the gold nanospheres (AuNSs) and nanosensors were characterized by the transmission electron microscope (TEM, Hitachi 600). The UV-vis absorption spectra of AuNSs, DNA and nanosensors were obtained with a Lambda 750 spectrophotometer (Perkin-Elmer). The zeta-potential of AuNSs and nanosensors were characterized by dynamic light scattering (DLS, Brookhaven 90 plus Zeta). The SERS measurements were carried out at 785 nm from a laser power of ∼7 mW and an accumulation time of 5 s each time.

**1.2 Preparation of nanosensor**

The AuNSs used in this work were prepared according to the classic citrate reduction reaction. In order to synthesize AuNSs-1, the DNA1 and aptamer sequences were annealed in phosphate buffer (8 mM) containing MgCl_2_ (0.8 mM) and NaCl (20 mM) to form double-strands. The annealed DNA strands were incubated in acetate buffer (pH = 5.4, 45 mM) with TCEP (1 mM) for 1.5 h to activate the thiol group. The DNA1/aptamer duplexes were then added dropwise to 10 nM citrate-capped AuNSs at a ratio of 1:60 (AuNSs:duplex), followed by salt aging for 24 h. For the preparation of AuNSs-2, the thiol groups in DNA2 single strands were activated as described for DNA1/aptamer duplexes, and then activated DNA2 and probe molecule DTNB (1 mM, 10 μL) were added to 10 nM AuNSs at a molar ratio of 1:100 (AuNSs:single strand), followed by salt aging for 24 h. The final AuNSs-1 and AuNPs-2 were purified by centrifugation at 15000 rpm for 10 min.

**1.3 SERS measurement of MMP-9 in solution**

The mixture solution of AuNSs-1 and AuNSs-2 (1:1 ratio) was incubated at 37℃ in a humidified incubator for 1 h. Then 20 μL of different concentrations of MMP-9 (0-350 ng/mL) were quickly added to 1.0 mL of the prepared AuNS mixture solution, and the reaction was performed at 37℃ for 2h for SERS detection. The laser wavelength and accumulation time used in this experiment are 785 nm and 5 s, respectively.

**1.4 Cell culture**

MDA-MB-231, Hs578bst, H8, HeLa, SW620 and MCF-7 cells were all bought from Shanghai ATCC Cell Bank, which has been granted permission by the Human Research Ethics Committee of the country for manipulations of human cells. They were grown in the Dulbecco’s Modifified Eagle’s Medium (DMEM) supplemented with 10% fetal bovine serum (FBS), 100 U/mL penicillin, and 100 μg/mL streptomycin at 37°C in a humidified atmosphere containing 5% CO_2_.

**1.5 Determination of MMP-9 from different types of cells**

Cells were cultured in glass-bottom dishes for 24 h in the complete cell culture medium. The supernatants of each cell line were collected and centrifuged three times (2000 rpm, 3 min) to remove cell debris and large protein. The mixture solution of AuNSs-1 and AuNSs-2 (1:1 ratio) was incubated at 37℃ in a humidified incubator for 1 h. Then 20 μL of the purified supernatants were added to 1.0 mL of the prepared AuNS mixture solution, and the reaction was performed at 37℃ for 2h for SERS detection. The SERS spectrum was obtained using a 785 nm laser and a collection time of 5 s.

1. **Quantification of DNA on each AuNSs**

The fluorescence spectroscopy is used to determine the amount of DNA on the gold sphere when AuNSs: DNA strand is in different proportions. The amount of DNA on AuNS-1 and AuNS-2 was quantified by measuring the fluorescence of DNA solution containing 1X SYBR Gold dye. The fluorescence intensity of the sample solution was measured with the excitation wavelength 485 nm and emission wavelength 528 nm. The average number of DNA strands per AuNS was calculated by dividing the concentration of DNAs by that of AuNSs.


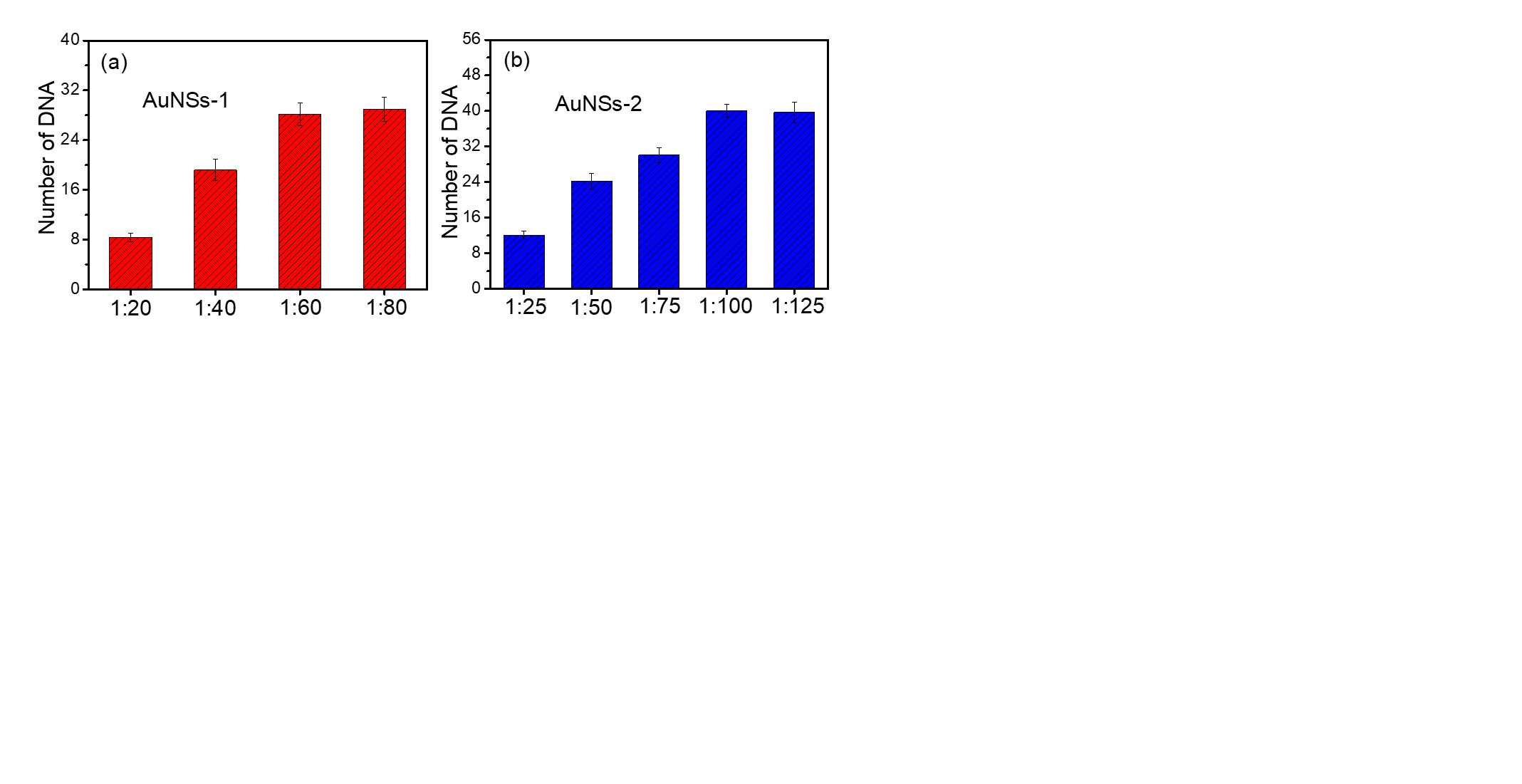


Fig. S1. DNA quantification on the surface of AuNSs-1 (a) and AuNSs-2 by adjusting the content ratio between AuNSs and DNA strand.

1. **Effect of MMP-9 concentration on hot spot formation**


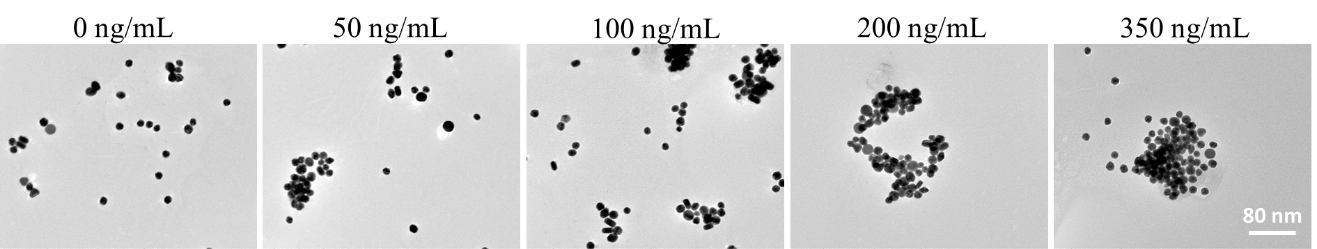


Fig. S2. TEM images of the nanosensor incubated with different concentrations of MMP-9.

1. **Optimization of the reaction time**

**
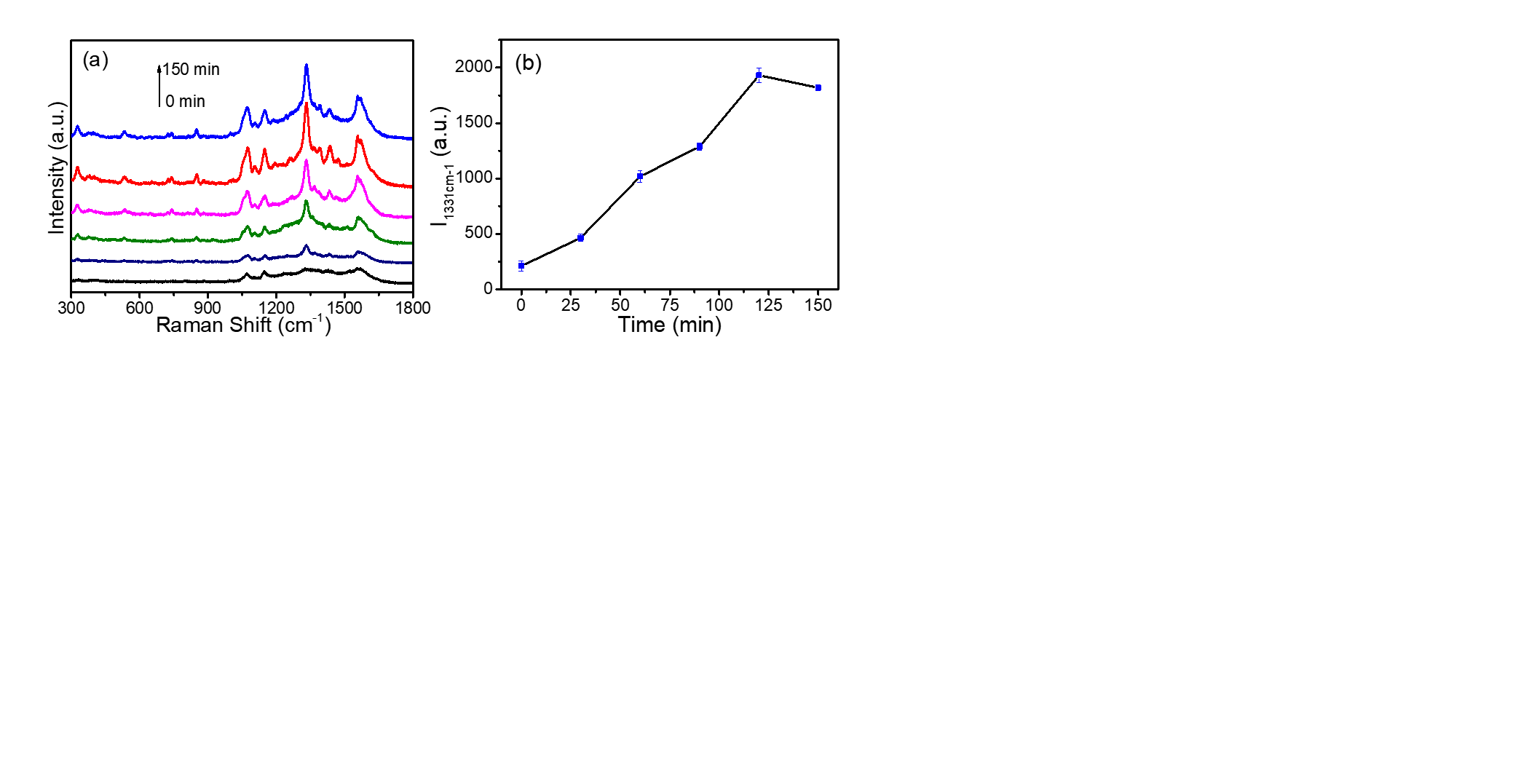
**

Fig. S3. (a) SERS spectra of the nanosensor at different reaction time (from bottom to top are 0, 30, 60, 90, 120 and 150 min, respectively) (b) A plot of the SERS intensity of the nanosensor at 1331 cm^-1^ along with reaction time.

1. **Optimization of the reaction temperature and pH**


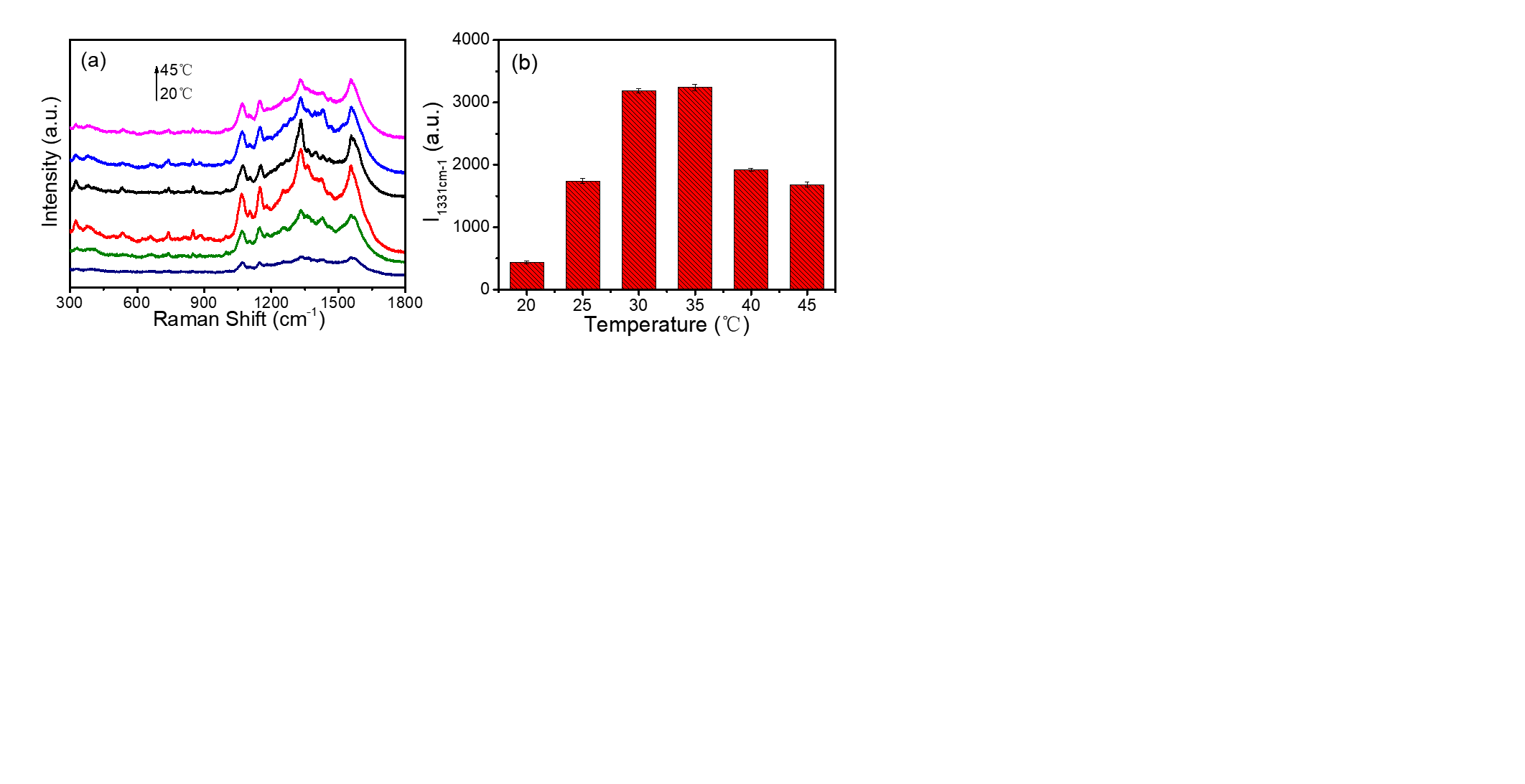


Fig. S4. (a) SERS spectra of the nanosensor at different temperatures (from bottom to top are 20, 25, 30, 35, 40, 45℃), while the reaction time kept at 120 min. (b) A plot of SERS intensity at 1331 cm^-1^ with the temperature changes.


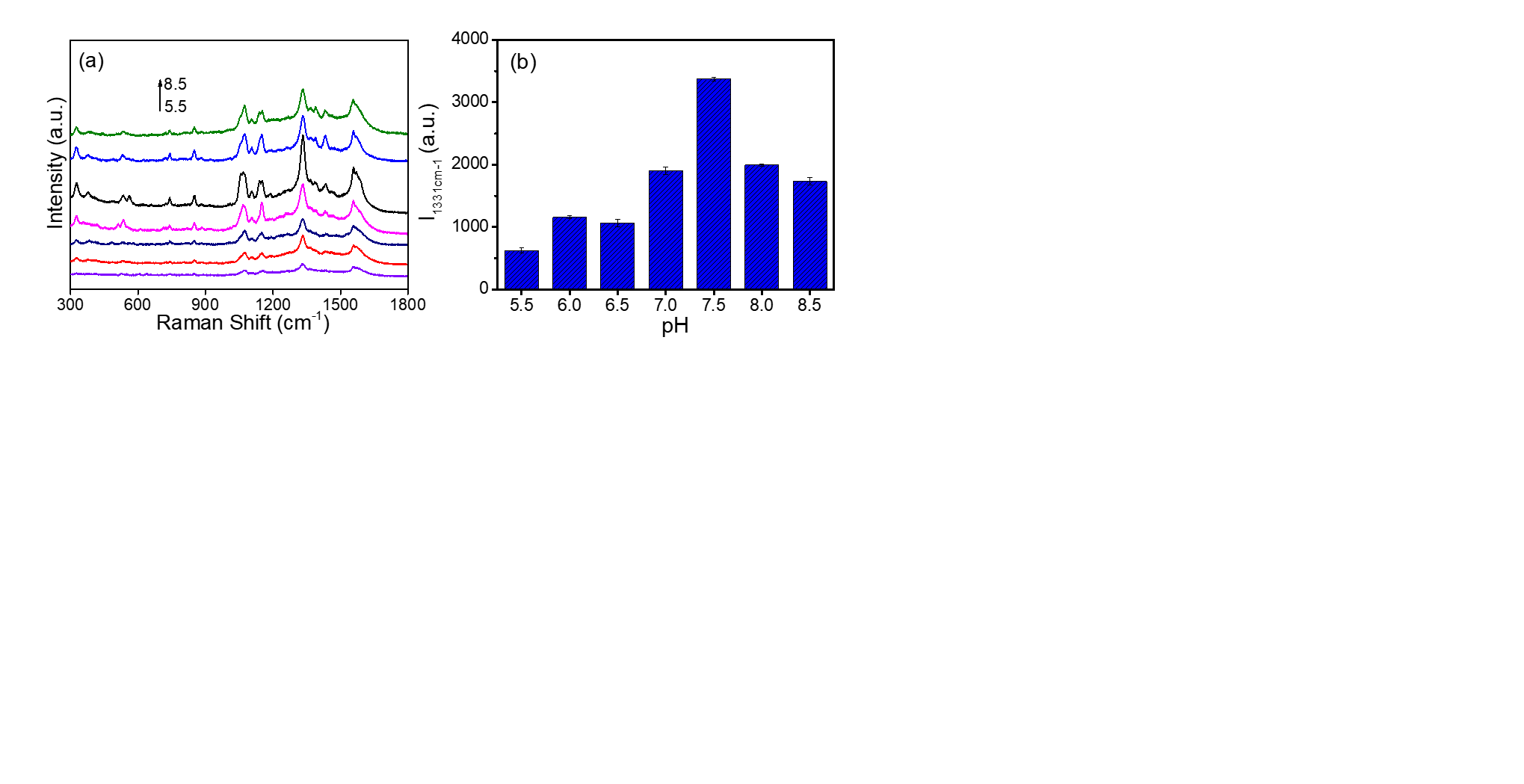


Fig. S5. (a) SERS spectra of the nanosensor at different pH (from bottom to top are 5.5, 6.0, 6.5, 7.0, 7.5, 8.0 and 8.5). (b) A plot of SERS intensity at 1331 cm^-1^ with the pH alteration.

1. **The evaluation of selectivity**


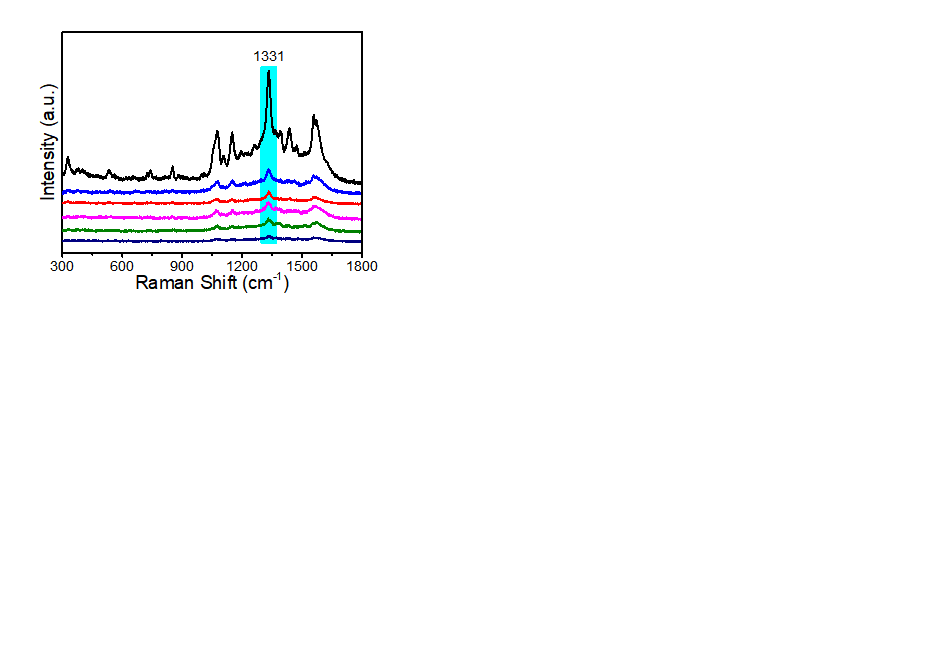


Fig. S6. SERS spectra studies of selectivity of this nanosensor with MMP-9 (10 ng/mL) and other interferents (100 ng/mL) (from the bottom to the top: blank, MMP-2, MMP-3, MMP-7, MMP-11 and MMP-9).


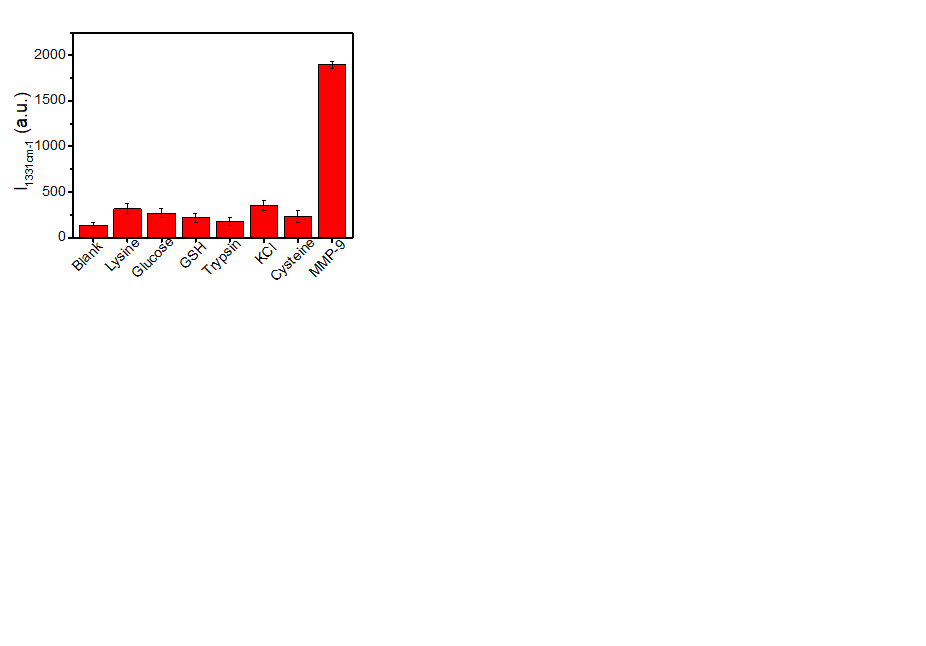


Fig. S7. SERS response of 1331 cm^-1^ to other biomolecules (100 ng/mL) and MMP-9 10 ng/mL).

1. **Stability test of the SERS nanosensor**


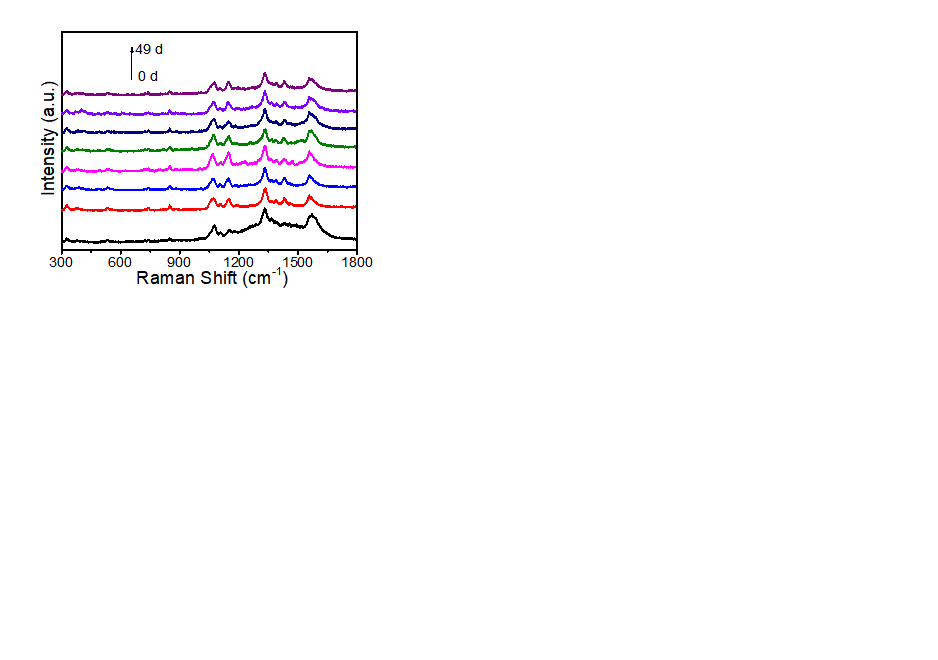


Fig. S8. SERS spectra of the nanosensor kept in natural conditions for different days (from bottom to top are 0, 7, 14, 21, 28, 35, 42 and 49 d).


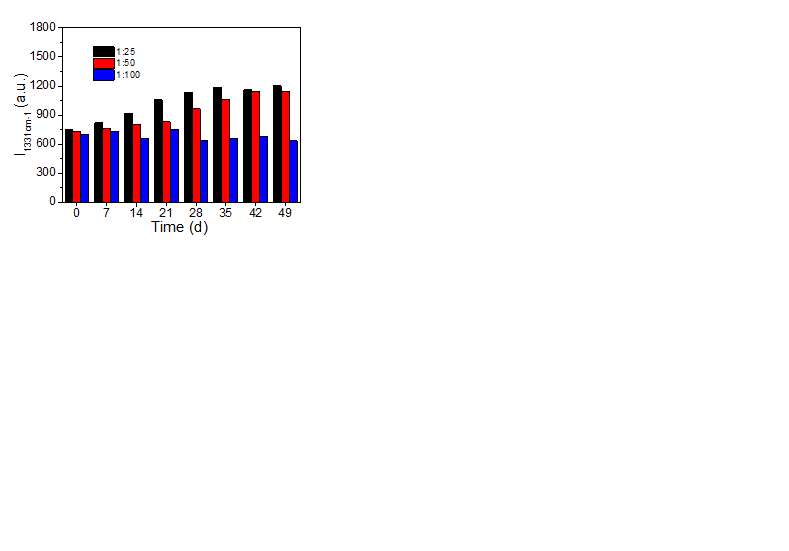


Fig. S9. The SERS intensity change of DTNB (1331 cm^-1^) located on the AuNSs-2 over time when the molar ratio of AuNSs:single is 1:25, 1:50, 1:100.

1. **Reproducibility of SERS detection**


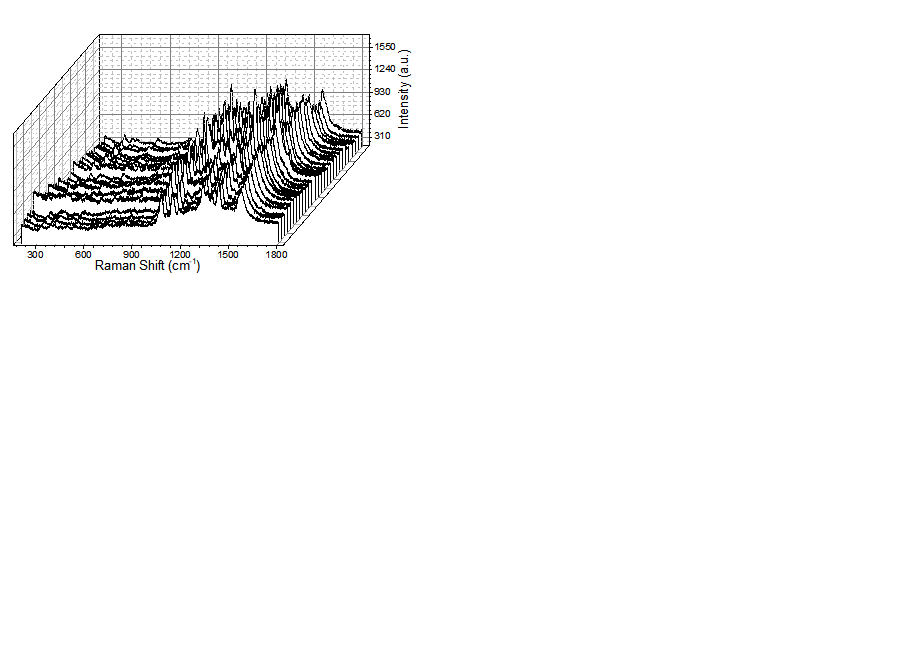


Fig. S10. SERS spectra from randomly selected 30 points on the same sample.

Table.S1 Comparisons of the proposed SERS nanosensor with other reported methods.

| Methods | Limit of detection | Detection range | Ref. |
| --- | --- | --- | --- |
| Electrochemical immunoassay | 0.92 ng/mL | 1-1000 ng/mL | [1] |
| FRET based method | 8 ng/mL | 16-140 ng/mL | [2] |
| Dual mode colorimetric and  fluorometric | 0.25 ng/mL | 1-200 ng/mL | [3] |
| Electrical dual-sensing method | 10 pM | 10 pM-10 nM | [4] |
| Electrochemical impedance  spectroscopy | 15 ng/mL | 50-400 ng/mL | [5] |
| SERS nanosensor | 0.2 ng/mL | 2-200 ng/mL | Ours |

Table.S2 The recovery rate of MMP-9 in the blood serum. Each sample was measured five times.

| Sample No. | Real value (ng/mL) | Measured value  (ng/mL) | Recovery (%) |
| --- | --- | --- | --- |
| 1 | 0.5 | 0.46±0.013 | 92±2.6 |
| 2 | 1.8 | 1.68±0.054 | 93.33±3.00 |
| 3 | 3.5 | 3.72±0.049 | 106.29±1.4 |
| 4 | 4.2 | 4.35±0.089 | 103.57±2.12 |
| 5  6 | 5.6  6.8 | 5.48±0.146  6.93±0.084 | 97.86±2.61  101.91±1.24 |

Table.S3 Assay results of detecting MMP-9 from serum samples using the SERS nanosensor and ELISA method.

| Sample No. | ELISA method (ng/mL) | SERS-based assay (ng/mL) |
| --- | --- | --- |
| 1 | 0.49±0.034 | 0.54±0.082 |
| 2 | 2.38±0.012 | 2.25±0.039 |
| 3 | 3.69±0.036 | 3.87±0.083 |
| 4 | 4.32±0.084 | 4.17±0.054 |
| 5  6 | 5.62±0.031  6.53±0.084 | 5.67±0.085  6.74±0.135 |
| 7 | 9.92±0.045 | 9.87±0.033 |

**References**

1. Shabani E, Abdekhodaie MJ, Mousavi SA, Taghipour F (2020) ZnO nanoparticle/nanorod-based label-free electrochemical immunoassay for rapid detection of MMP-9 biomarker. Biochem Eng J 164: 107772.

2. Synak A, Serdiuk I, Grobelna B, Fudala R, Gryczynski I, Bojarski P (2019) Spectroscopic method for estimation of MMP-9 enzyme concentration and activity. J Mol Liq 286: 110936.

3. Dadmehr M, Mortezaei M, Korouzhdehi B (2023) Dual mode fluorometric and colorimetric detection of matrix metalloproteinase MMP-9 as a cancer biomarker based on AuNPs@gelatin/AuNCs nanocomposite. Biosens Bioelectron 220: 114889.

4. Tran TB, Nguyen PD, Baek C, Min JH (2016) Electrical dual-sensing method for real-time quantitative monitoring of cell-secreted MMP-9 and cellular morphology during migration process. Biosens Bioelectron 77: 631–637.

5. Biela A, Watkinson M, Meier UC, Baker D, Giovannoni G, Becer CR, Krause S (2015) Disposable MMP-9 sensor based on the degradation of peptide cross-linked hydrogel films using electrochemical impedance spectroscopy. Biosens Bioelectron 68: 660–667.
